# 

**Published:** 1897-12

**Authors:** 


### CONTENTS.

### COMMUNICATIONS.

Replantation in Pyorrhea Alveolaris ........ 569
Report of a Case Verifying the Statement First Made by Dr. M. H. Cryer, Showing Direct Communication of the Frontal Sinus with the Antrum of Highmore ........ 573
An Obscure Case ........ 574
Opening the Bite with Cap-Fillings, Preserving the Vitality of the Pulp ........ 576
The Amalgam Question ........ 577
Structural Development ........ 578
The Compatibility of Different Filling Materials with Tooth Structure ........ 579
The Relations between the Oral Cavity and the General System ........ 582
Soldering Aluminum ........ 583
What is the Best Method for Teaching Children to care for their Teeth? ........ 586

### PROCEEDINGS.

Michigan Dental Association ........ 589
The Evansville Dental Society ........ 596
Northern Illinois Dental Society ........ 597

### SELECTIONS.

Applications to Produce Local Anesthesia ........ 572
Simple Rule for Ascertaining the Length of Days and Nights ........ 585
Wroblewski's Local Anesthetic ........ 595
Paracelsus on Doctors ........ 598
A New Dressing ........ 598
The Labors of State Boards of Medical Examiners and Licensers and the State and other Boards of Health ........ 599
Pain and its Therapeusis ........ 600
Longevity ........ 600
Properties and Uses of Papain ........ 601
The Chin as an Index of Character ........ 602
Fossil Bacteria ........ 603
First International Exhibition of Dentistry and Dental Surgery ........ 604
Headaches from Eye Strain ........ 604
Nothing but Luck ........ 605

### NOTICES.

National Association of Dental Technics ........ 605
The Southern Dental Association ........ 605

### EDITORIAL.

Bibliographical—Tin Foil and its Combinations for Filling Teeth ... 606
Aluminum Lined Rubber Plates ........ 607

### BIOGRAPHICAL.

Dr. Thomas W. Evans ........ 607
Dr. D. R. Jennings ........ 609
Dr. W. W. Sheffield ........ 610

